# Profile and Functional Prediction of Plasma Exosome-Derived CircRNAs From Acute Ischemic Stroke Patients

**DOI:** 10.3389/fgene.2022.810974

**Published:** 2022-03-14

**Authors:** Jie Yang, Junli Hao, Yapeng Lin, Yijia Guo, Ke Liao, Min Yang, Hang Cheng, Ming Yang, Kejie Chen

**Affiliations:** ^1^ Department of Neurology, Sichuan Provincial People’s Hospital, University of Electronic Science and Technology of China, Chengdu, China; ^2^ Department of Neurology, Clinical Medical College, The First Affiliated Hospital of Chengdu Medical College, Chengdu, China; ^3^ School of Bioscience and Technology, Chengdu Medical College, Chengdu, China; ^4^ International Clinical Research Center, Chengdu Medical College, Chengdu, China; ^5^ School of Public Health, Chengdu Medical College, Chengdu, China

**Keywords:** circRNA, plasma exosome, profile, acute ischemic stroke, ceRNA

## Abstract

Stroke is one of the major causes of death and long-term disability, of which acute ischemic stroke (AIS) is the most common type. Although circular RNA (circRNA) expression profiles of AIS patients have been reported to be significantly altered in blood and peripheral blood mononuclear cells, the role of exosome-containing circRNAs after AIS is still unknown. Plasma exosomes from 10 AIS patients and 10 controls were isolated, and through microarray and bioinformatics analysis, the profile and putative function of circRNAs in the plasma exosomes were studied. A total of 198 circRNAs were differentially quantified (|log2 fold change| ≥ 1.00, *p* < 0.05) between AIS patients and controls. The levels of 12 candidate circRNAs were verified by qRT-PCR, and the quantities of 10 of these circRNAs were consistent with the data of microarray. The functions of host genes of differentially quantified circRNAs, including RNA and protein process, focal adhesion, and leukocyte transendothelial migration, were associated with the development of AIS. As a miRNA sponge, differentially quantified circRNAs had the potential to regulate pathways related to AIS, like PI3K-Akt, AMPK, and chemokine pathways. Of 198 differentially quantified circRNAs, 96 circRNAs possessing a strong translational ability could affect cellular structure and activity, like focal adhesion, tight junction, and endocytosis. Most differentially quantified circRNAs were predicted to bind to EIF4A3 and AGO2—two RNA-binding proteins (RBPs)—and to play a role in AIS. Moreover, four of ten circRNAs with verified levels by qRT-PCR (hsa_circ_0112036, hsa_circ_0066867, hsa_circ_0093708, and hsa_circ_0041685) were predicted to participate in processes of AIS, including PI3K-Akt, AMPK, and chemokine pathways as well as endocytosis, and to be potentially useful as diagnostic biomarkers for AIS. In conclusion, plasma exosome-derived circRNAs were significantly differentially quantified between AIS patients and controls and participated in the occurrence and progression of AIS by sponging miRNA/RBPs or translating into proteins, indicating that circRNAs from plasma exosomes could be crucial molecules in the pathogenesis of AIS and promising candidates as diagnostic biomarkers and therapeutic targets for the condition.

## Introduction

As a common vascular disease, stroke is one of the major causes of death and long-term disability, challenging the current healthcare system worldwide (Hu et al., 2017; Szegedi et al., 2017). Of all stroke cases, ischemic stroke (IS) accounts for more than 80% of them (Benjamin et al., 2018). In humans, IS results from various factors or diseases, including hypertension, large-vessel atherosclerosis and rupture of an atherosclerotic plaque, cardioembolism, and lacunar infarcts induced by small-vessel disease. However, the underlying mechanism and relationship of these factors or diseases with stroke remain incompletely known, which restricts safer and more efficient treatment to stroke. To treat patients with IS, early reperfusion of blocked arteries is most frequently performed, even though it is constrained by a narrow time window and risk of bleeding (Snow, 2016; Alberts, 2017; Powers et al., 2018). Therefore, novel targets for diagnostics or therapeutics are urgently needed for patients with IS.

Exosomes, nanosized vesicles (∼30–150 nm) with lipid bilayer membranes, are secreted by various cells and naturally present in the blood (Caby et al., 2005; Couzin, 2005; Raposo and Stoorvogel, 2013). When incorporating into target recipient cells, exosomes release cargos, like proteins, lipids, DNA, mRNAs, and noncoding RNAs (Hon et al., 2017; Skotland et al., 2017; Mousavi et al., 2019), into the cellular microenvironment of recipient cells (Cocucci et al., 2009; Simons and Raposo, 2009). Therefore, exosomes play a crucial role in cell-to-cell communication and intercellular signal transduction (Zhang et al., 2017) and are emerging as promising targets for the pathogenesis of diseases and potent tools for diagnosis and therapy. Among the various cargos in exosomes, circular RNA (circRNA) is attracting more attention due to its unique structure. CircRNAs are a class of noncoding RNAs with covalent bonds between 3ʹ head and 5ʹ tail ends to produce a circular pattern (Memczak et al., 2013), a more stable one than that of linear RNAs (Suzuki et al., 2006). Moreover, circRNAs are highly conserved and abundantly expressed in human cells (Rybak-Wolf et al., 2015) and characterized by distinct tissue-specific expression (Memczak et al., 2013). The high stability, evolutionary conservation, and abundance of circRNAs in various species endow them with numerous different potential functions in the regulation of gene expression, such as miRNA sponges, interactions with RNA-binding proteins (RBPs), or translation into proteins (Han et al., 2018). Consequently, circRNAs have been considered important biological regulators for understanding the molecular mechanisms of disease and identifying effective diagnostic biomarkers or therapeutic targets (Liu W. et al., 2017).

CircRNAs have been reported to be involved in the progression of many diseases, including Alzheimer’s disease (Lukiw, 2013), cardiovascular diseases (Du et al., 2017a; Jiang et al., 2019), atherosclerosis (Burd et al., 2010), stroke (Peng et al., 2019; Li et al., 2021), and various cancers (Li et al., 2015; Yao et al., 2017). Recently, circRNAs have been considered to be potential biomarkers or targets for diagnosis or treatment of acute IS (AIS) (Lu et al., 2020; Zuo et al., 2020). CircUCK2, as a miRNA sponge, markedly reduced IS infarct volume and improved neurological impairment in the mouse model of focal cerebral ischemia and reperfusion (Chen W. et al., 2020), while circHECTD1 aggravated cerebral infarction volume and neuronal apoptosis in the mouse model of IS (Dai et al., 2021). Moreover, circRNAs exhibit dynamic expression patterns in a series of physiological and pathological conditions and possess important regulatory effects. Expression profiles of circRNAs from peripheral blood mononuclear cells and blood of patients with AIS are significantly different from those of controls (Li S. et al., 2020; Dong et al., 2020; Ostolaza et al., 2020).

However, the profile and regulatory network of circRNAs derived from plasma exosomes of patients with AIS remain unclear. In this prospective investigation, a case-controlled study was conducted, and the circRNA profile of plasma exosomes was compared between AIS patients and controls through microarray. By bioinformatics approaches, the functions and signaling pathways of differentially quantified circRNAs were analyzed. Based on the results of microarray, 12 circRNAs with high fold changes among differentially quantified circRNAs were chosen for validation by qRT-PCR, and their roles in the pathogenesis of AIS and diagnostic values for AIS were also explored. The data of the present study shed light on the possible roles of exosome-derived circRNAs in regulating the pathogenesis of AIS and provide new potential targets for diagnosis and therapy of AIS.

## Materials and Methods

### Study Subjects

Ten adult patients with AIS (five males and five females) were selected from the First Affiliated Hospital of Chengdu Medical College in 2020. AIS was diagnosed according to the Guidelines for the Prevention of Stroke in Patients with Stroke and Transient Ischemic Attack (Kernan et al., 2014). Ten controls approximately age-matched were enrolled from the health management center of the First Affiliated Hospital of Chengdu Medical College in 2020. The demographic description of AIS patients and controls is shown in Table 1. The investigation was approved by the Ethical Committee of First Affiliated Hospital of Chengdu Medical College. All study participants or their authorized representatives signed informed consent forms.

**TABLE 1:** Demographic characteristics of AIS patients and controls.

Characteristics	AIS patients	Controls
Age (mean ± SD), year	75.80 ± 7.84	68.17 ± 10.97
Male, *n* (%)	5 (50%)	5 (50%)
Ethnic Han, *n* (%)	10 (100%)	10 (100%)
Time after onset (mean ± SD), hour	6.65 ± 4.37	—
Comorbidity, n (%)		
Hypertension	4 (40%)	0 (0%)
Diabetes	3 (30%)	0 (0%)
Coronary heart disease	4 (40%)	0 (0%)
Atrial fibrillation	2 (20%)	0 (0%)

—, not applicable.

Inclusion criteria are as follows: (1) aged 50–90 years old, (2) within 24 h after onset, and (3) have agreed to sign the informed consent. Exclusion criteria are as follows: (1) have received thrombolysis and/or thrombectomy treatment and (2) have other concomitant diseases, including acute infection, immune diseases, neurodegenerative diseases, and tumors.

### Isolation and Characterization of Plasma Exosomes

Blood samples from 10 AIS patients and 10 controls included in the present study were collected into EDTA tubes by venipuncture. After centrifuging at 2,000 rcf for 15 min at room temperature, a clear top layer was harvested to a labeled tube (BS-50-M, Biosharp, China) and stored at −80°C. Plasma exosomes were isolated using the exoRNeasy Serum/Plasma Maxi kit (77064, Qiagen Sciences Inc., Germany) following the manufacturer’s instruction. The resulting exosome pellet was suspended in PBS for further RNA or protein extraction. The exosome fraction isolated from plasma was characterized by western blot, nanoparticle tracking analysis (NTA), and transmission electron microscopy (TEM).

#### Western Blot

Plasma exosomes suspended in PBS were mixed with the same volume of RIPA lysis buffer, and exosome proteins were purified from each sample and separated by SDS-PAGE gel and then transferred to a polyvinylidene fluoride (PVDF) membrane. The membrane was blocked using 5% BSA and then treated with antibodies against CD63 (ab134045, Abcam, United Kingdom) or TSG101 (ab133586, Abcam, United Kingdom) at 4°C for 12 h. Horseradish peroxidase-conjugated secondary antibody (SBI, United States) was then applied, and the blots were developed with enhanced chemiluminescence reagents.

#### NTA

Isolated exosomes were diluted in PBS and analyzed using the ZetaView (S/N 17-310, Particle Metrix, Germany) with NTA software (ZetaView 8.04.02). Triplicate measurements were recorded for each sample. Size distribution and concentration profiles were averaged to derive the representative size distribution profiles.

#### TEM

Plasma exosomes suspended in PBS were fixed in 2% paraformaldehyde. The fixed sample was absorbed onto formvar-coated copper grids for 5 min at room temperature. After being rinsed in distilled water, samples were stained with methyl cellulose uranyl acetate for 1 min at room temperature. Excess liquid was wicked off the grid using filter paper, and grids were stored at room temperature until imaging. Imaging was performed using Tecnai G2 Spirit BioTwin (FEI, United States).

### Total RNA Extraction From Plasma Exosomes

By exoRNeasy Serum/Plasma Maxi Kit (77064, Qiagen Sciences Inc., Germany), total RNA was extracted from plasma exosomes isolated from the 10 AIS patients and 10 controls following the manufacturer’s recommendation. RNA integrity of total RNA was inspected by an Agilent Bioanalyzer 4200 (Agilent Technologies, Santa Clara, CA, United States) (data not shown).

### CircRNA Microarray Profiling

SurePrint G3 Human ceRNA microarray (4 × 180K, design ID: 085499) was made by Agilent Technologies and contained probes interrogating 84,569 circRNAs. Total RNAs were incubated with RNase R to eliminate linear RNAs. The enriched circRNAs were transcribed into fluorescent complementary RNA (cRNA), which then were hybridized onto the circRNA microarray. After hybridization, the signal of the circRNA microarray was scanned using the Agilent Microarray Scanner (Agilent Technologies Inc., Santa Clara, CA, United States).

### Microarray Data Analysis

The scanned information was extracted using the Agilent Feature Extraction software 10.7 (Agilent Technologies Inc., Santa Clara, CA, United States). Raw data were normalized by quantile algorithm, R package “limma”. Differentially quantified circRNAs were identified through volcano plot filtering. Hierarchical clustering analysis was conducted to display the circRNA profile pattern between patients with AIS and controls.

### Validation of Selected Differentially Quantified circRNAs by qRT-PCR

The microarray results were verified by qRT-PCR using samples from the same subjects (10 AIS patients and 10 controls). Total RNA from plasma exosomes was incubated with RNase R to eliminate linear RNAs and reverse transcribed by RevertAid First-Strand cDNA Synthesis Kit (K1622, Thermo Scientific, United States). CircRNA levels were determined by qRT-PCR *via* TB Green^®^ *Premix Ex Taq*™ II (RR820Q, TaKaRa, Japan) with the BIO-RAD system (MiniOpticon, United States) according to the instruction of the manufacturer. The reaction system was 25 μl in volume and consisted of 12.5 μl of TB Green *Premix Ex Taq* II, 2 μl of cDNA, 1 μl of forward and reverse primers (10 μM), and 8.5 μl of RNase-free water. The optimum reaction conditions were as follows: 95°C for 30 s, followed by 40 cycles of 95°C for 5 s and 60°C for 30 s. Twelve circRNAs with high fold changes from the microarray results were chosen for validation by qRT-PCR with GAPDH as a normalization reference. The specific primers for target circRNAs are described in Table 2 and synthesized by Sangon Biotech (Shanghai, China). The primers for GAPDH were purchased from Sangon Biotech (B662104, Sangon Biotech, China). All reactions were performed in triplicate. The threshold cycle (Ct) was determined using the default threshold settings, and the average Ct value was used to calculate the relative fold changes of the 12 circRNAs by the 2^−ΔΔCt^ method (Livak and Schmittgen, 2001).

**TABLE 2:** The primer sequences of 12 differentially quantified circRNAs.

circRNA	Sequence (5′–3′)
hsa_circ_0131433	Forward: TCT​GTG​AGG​TTC​TTG​ATT​TGG​A
	Reverse: TCT​TTT​CCC​TCT​TGC​CTT​CC
hsa_circ_0123103	Forward: GAA​ATG​CCG​TGT​GGA​ACT​CT
	Reverse: TTC​ATG​ATC​ACT​TGG​GCA​GT
hsa_circ_0112036	Forward: CAC​GAG​AAT​TGA​AGT​GGG​AGA
	Reverse: TGC​ATT​TCG​AGA​GCA​ATG​AG
hsa_circ_0050840	Forward: CTT​GTC​CAC​TCC​GGC​TAA​AG
	Reverse: CTC​CTG​TTC​ATG​TGG​GGA​CT
hsa_circ_0077256	Forward: TTG​AAT​GAG​GGT​CGC​TGT​CT
	Reverse: AGC​TGC​TAG​TCA​GTC​ACA​TTC​G
hsa_circ_0113001	Forward: GAC​CAG​GCA​AGC​TAG​TGC​TC
	Reverse: TTT​GGA​GCA​CTC​TTC​AAG​TCC
hsa_circ_0092545	Forward: GAA​ATG​GCA​ACT​TTC​CTC​CA
	Reverse: CCA​CAC​AGC​ATC​AGG​TTT​TG
hsa_circ_0041685	Forward: GAC​AGT​CTC​CAG​GGA​AAG​CA
	Reverse: GCC​TCC​TGA​ATC​TGA​ATT​CCT
hsa_circ_0059662	Forward: ATC​TCC​TGT​CCC​CTG​CTC​AT
	Reverse: TGA​GTC​ACC​CCA​ACC​TCT​GT
hsa_circ_0032222	Forward: ACC​TGT​GTG​GCC​TGG​TAC​AT
	Reverse: GTG​TCA​ATG​GCA​TCC​TCC​AC
hsa_circ_0093708	Forward: AAC​ACA​GCT​GAC​TGG​GTC​CT
	Reverse: CAG​CCT​CTC​TTC​TCC​AGG​AA
hsa_circ_0066867	Forward: CCT​GTT​GGT​TGC​TCT​TTT​CA
	Reverse: TGA​TAG​GTG​GGA​CTG​GAA​GG

### Bioinformatics Analysis and Target Prediction

Host/target genes of differentially quantified circRNAs were illuminated by Gene Ontology (GO, http://www.geneontology.org/) and Kyoto Encyclopedia of Genes and Genomes (KEGG, http://kobas.cbi.pku.edu.cn/) analyses. Target miRNAs of circRNAs were predicted using miRanda (http://www.microrna.org/microrna/home.do) and TargetScan (http://www.targetscan.org/vert_72/). MiRNA response elements as regulators of mRNAs were predicted through TargetScan (http://www.targetscan.org/vert_72/) and microT-CDS (http://www.microrna.gr/microT-CDS). All circRNA–miRNA–mRNA interaction networks of differentially quantified circRNAs were visualized by Cytoscape 3.8.2. The tissue specificity of differentially quantified circRNAs was annotated *via* TSCD (http://gb.whu.edu.cn/TSCD/). The types and sites of RBPs binding to differentially quantified circRNAs were annotated by circinteractome (https://circinteractome.nia.nih.gov/). The probability of differentially quantified circRNAs coding for proteins was estimated by circBank (http://www.circbank.cn/), in which circRNAs with a coding probability >0.99 were analyzed.

### Receiver Operating Characteristic Curve Analysis

The levels of differentially quantified circRNAs verified by qRT-PCR were applied to generate ROC curves for the 10 AIS patients and 10 controls. The area under the curve (AUC) was calculated to assess the predictive value of the selected circRNAs for AIS diagnosis.

### Statistical Analysis

The differentially quantified circRNAs were defined as |log_2_ fold change| ≥ 1.00 and *p* < 0.05. The *p*-value denotes the significance of GO and KEGG pathway analyses (*p* < 0.05). All statistical analyses were performed by SPSS (26.0, IBM, United States). All data were represented as the mean ± standard deviation ( $\bar{\text{X}}$ ± SD). A *p*-value <0.05 was considered statistically significant.

## Results

### Characterization of Plasma Exosome

**FIGURE 1:**
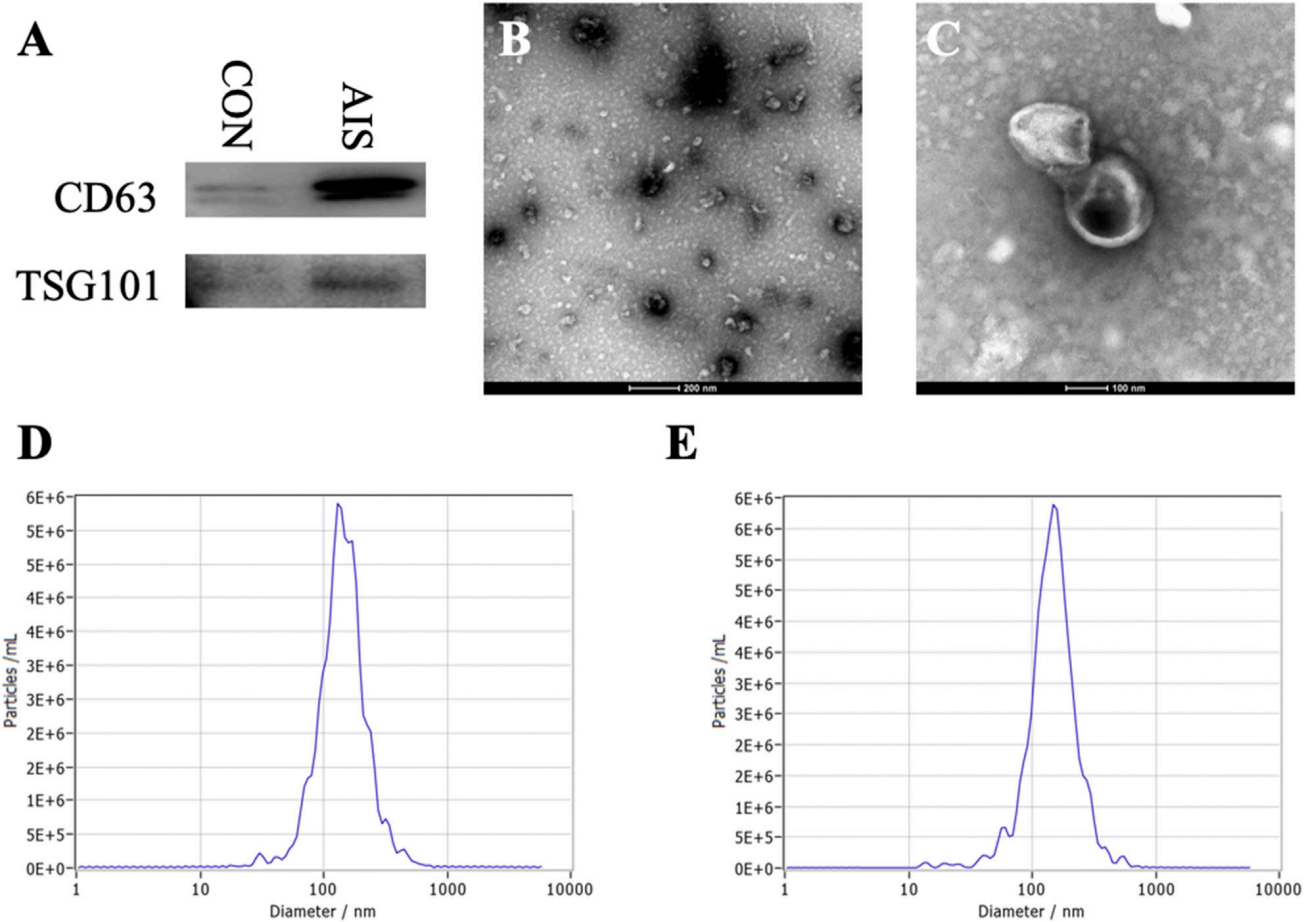
Identification and characterization of plasma exosomes. **(** **A** **)** Detection of CD63 and TSG101 as exosome markers by western blot. **(** **B**,**C** **)** Representative images of transmission electron microscopy. **(** **D**,**E** **)** Representative size of exosomes from controls and AIS patients, respectively.

The exosomes isolated from plasma of patients with AIS and controls had obvious detectable levels of CD63 and TSG101 by western blot (Figure 1A). The typical bilayer of exosomes was observed (Figures 1B,C) through TEM, and the median size of exosomes isolated from plasma was ∼137 nm for controls and ∼141 nm for patients with AIS (Figures 1D,E, respectively). These data confirmed that we successfully isolated plasma exosomes from blood.

### Profile of CircRNAs Derived From Plasma Exosomes

**FIGURE 2:**
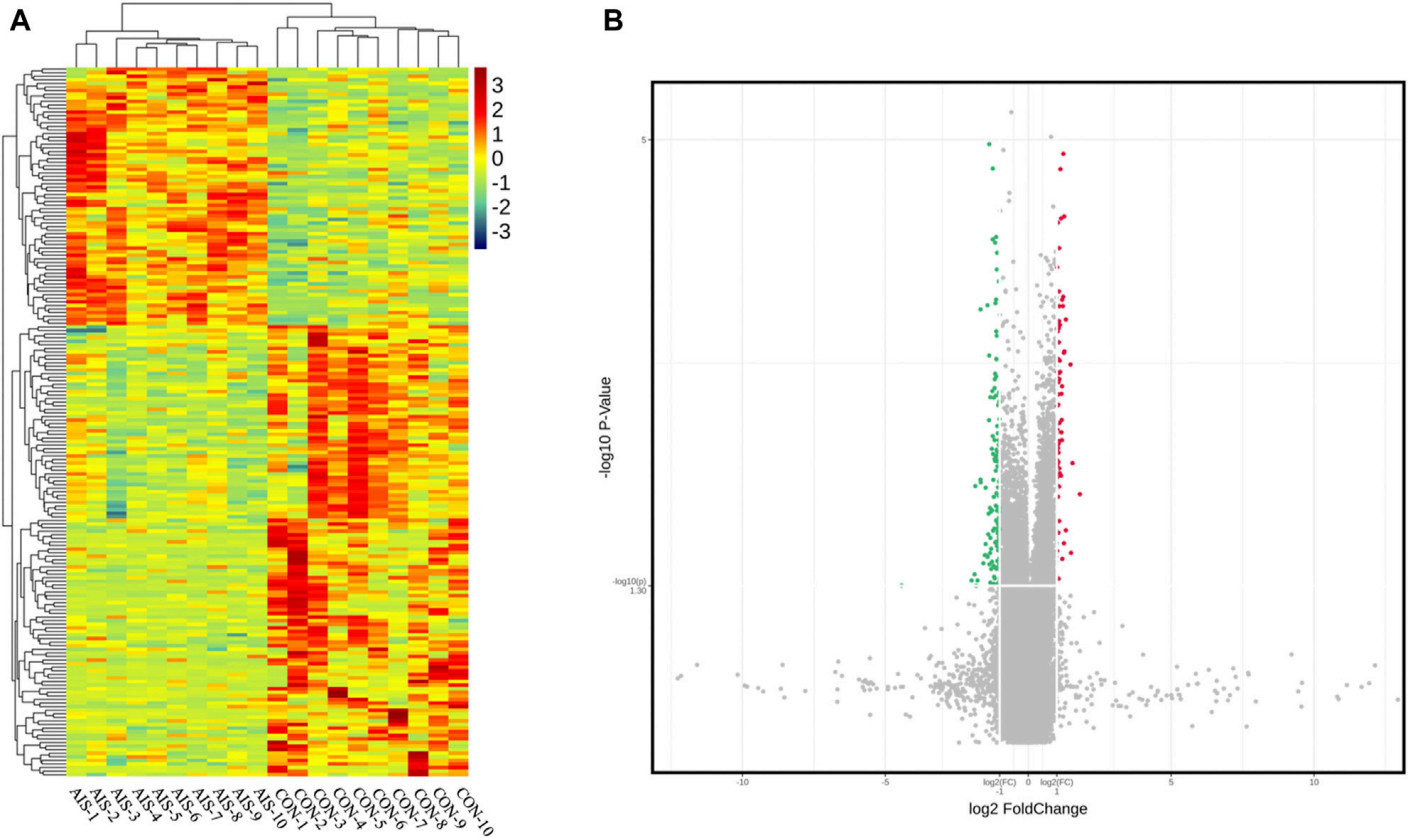
CircRNA profiles of plasma exosomes from AIS patients (AIS) and controls (CON). **(A)** Heat map of circRNAs differentially quantified between AIS patients and controls. **(B)** Volcano plot of circRNA profiles between AIS patients and controls. Red dots represent significantly upregulated circRNAs (log_2_ fold change ≥ 1.00 and *p* < 0.05, *n* = 10, independent two-sample *t*-test), and green dots represent significantly downregulated circRNAs (log_2_ fold change ≤ −1.00 and *p* < 0.05, *n* = 10, independent two-sample *t*-test).

The profile of 84,569 human circRNAs was generated by SurePrint G3 Human ceRNA microarray using plasma exosomes from patients with AIS and controls. After fold-change filtering, 198 circRNAs were differentially quantified between patients with AIS and controls, including 72 upregulated (log_2_ fold change ≥ 1.00, *p* < 0.05) and 126 downregulated (log_2_ fold change ≤ −1.00, *p* < 0.05) circRNAs. Hierarchical clustering of the levels between AIS patients and controls is illustrated in Figure 2A. The volcano plot showed that significant variation was observed in circRNA levels between AIS patients and controls (Figure 2B).

**FIGURE 3:**
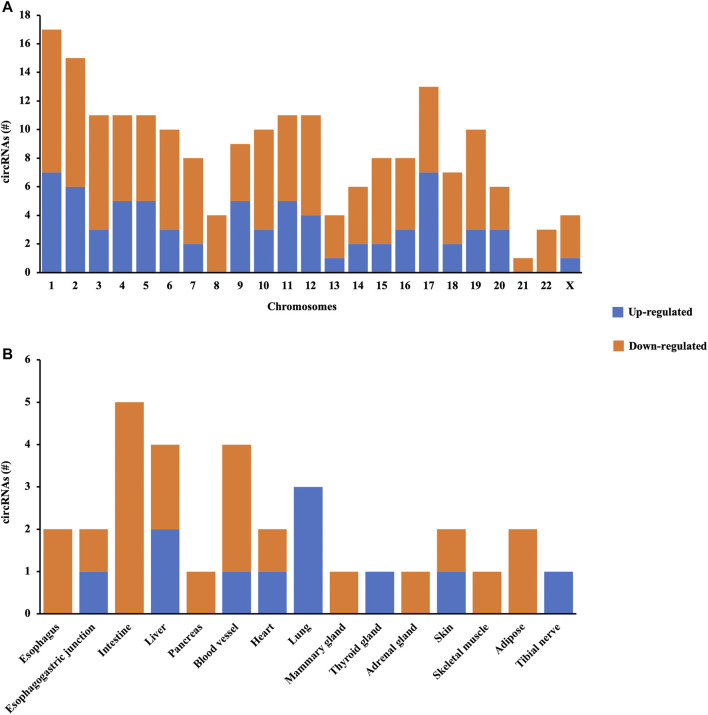
Characterization of circRNA profiles between AIS patients and controls. **(A)** Bar diagram of the distribution of differentially quantified circRNAs in different chromosomes. **(B)** Top 15 tissue-specific distributions of differentially quantified circRNAs.

The distribution patterns of differentially quantified circRNAs in the chromosomes are shown in Figure 3A, indicating that the profile of circRNAs in patients with AIS was significantly different from that in controls. Moreover, the tissue-specific analysis showed that the differentially quantified circRNAs were mainly derived from the digestive system (esophagus, esophagogastric junction, intestine, and liver), the cardiovascular system (blood vessel and heart), and lungs (Figure 3B).

### GO and KEGG Pathway Analyses of Host Genes of Differentially Quantified circRNAs

**FIGURE 4:**
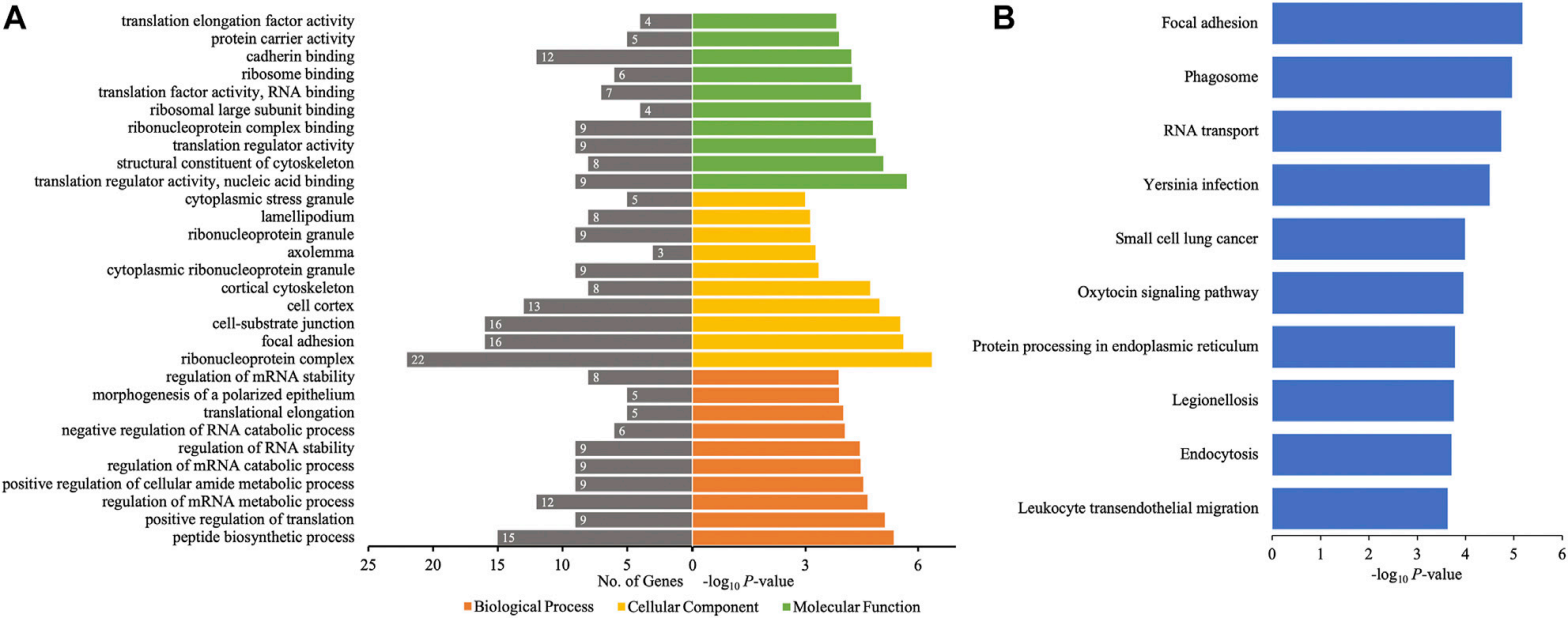
GO annotations and KEGG pathway analyses for host genes of the differentially quantified circRNAs. **(A)** GO annotations. The GO analysis categorized the mRNAs into different groups under the theme of biological process, cellular component, and molecular function. The gene count for each GO category is listed on the left. **(B)** Top 10 KEGG pathways. The vertical axis indicates the pathway category, and the horizontal axis indicates the −log_10_ *p*-value of a pathway (Fisher’s exact test).

The different profiles of circRNAs between patients with AIS and controls could be the result of disordered expression of host genes generating circRNAs; thus, GO and KEGG pathway analyses were performed to evaluate the putative functions of host genes. According to the GO analysis shown in Figure 4A, the host genes of differentially quantified circRNAs, in the cellular component, were mainly involved in ribonucleoprotein (ribonucleoprotein complex, cytoplasmic ribonucleoprotein granule, and ribonucleoprotein granule) and cell junction (focal adhesion and cellsubstrate junction). The molecular functions related to host genes mainly included translation regulator activity and binding (ribosome, ribonucleoprotein, and cadherin), and the major biological processes of host genes were associated with RNA regulation. The KEGG analysis showed that intracellular physiological processes (like RNA transport and protein processing in endoplasmic reticulum) and intercellular interaction (like focal adhesion and leukocyte transendothelial migration) could be affected, indicating cellular functions disrupted in AIS (Figure 4B).

### Validation of Levels of Differentially Quantified circRNAs

**FIGURE 5:**
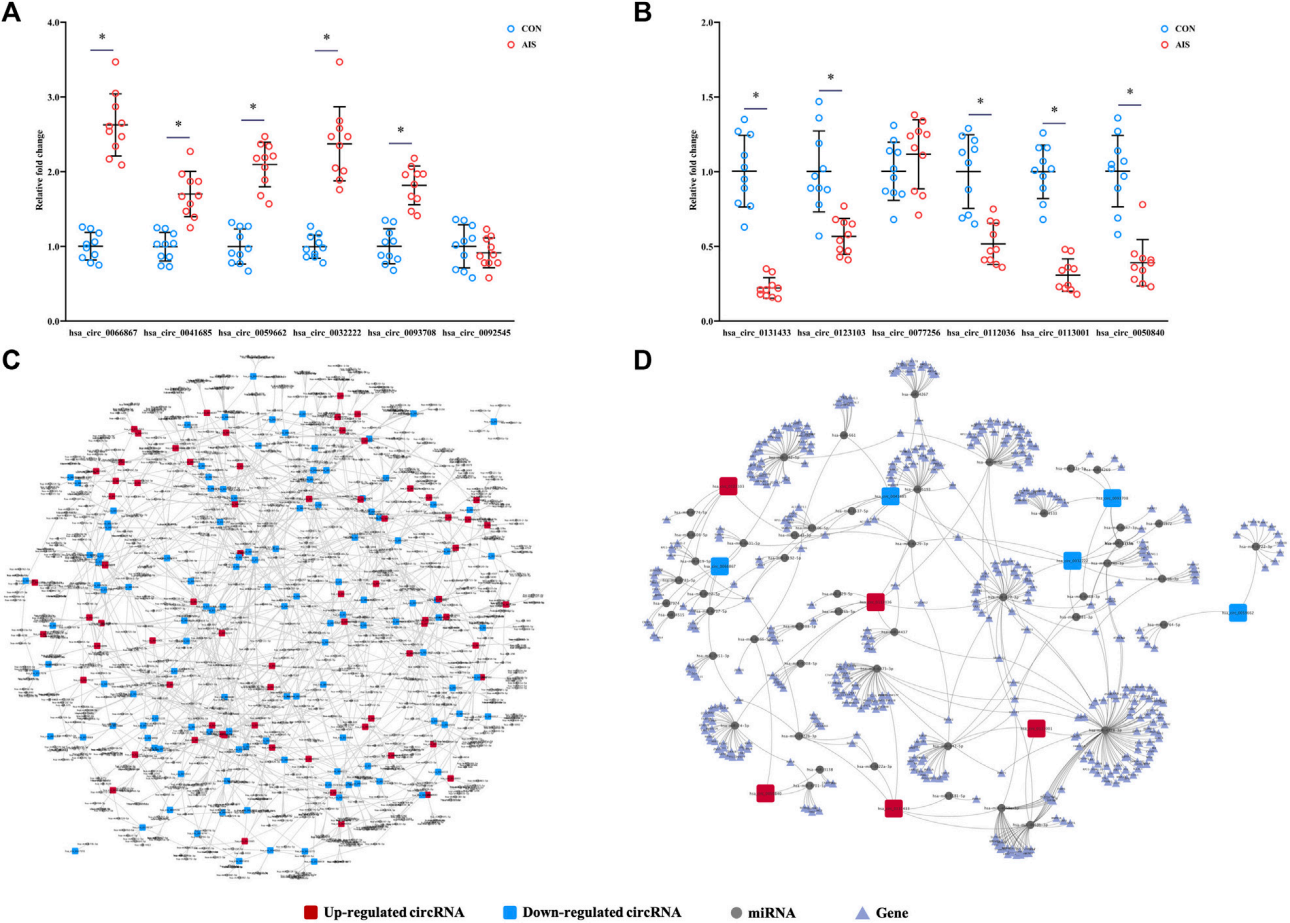
Validation and network diagram of differentially quantified circRNAs. **(** **A**,**B** **)** Relative fold changes of 12 differentially quantified circRNAs using qRT-PCR (*n* = 10). *, *p* < 0.05. **(** **C** **)** Network of circRNA-miRNA of 198 differentially quantified circRNAs. **(** **D** **)** Network of circRNA-miRNA-mRNA of the 10 differentially quantified circRNAs verified by qRT-PCR.

To validate the results from microarray, the levels of 12 differentially quantified circRNAs with high fold changes were determined using qRT-PCR. Among 12 selected differentially quantified circRNAs, the levels of 10 circRNAs were significantly different (*p* < 0.05) between AIS patients and controls, including five upregulated (hsa_circ_0066867, hsa_circ_0093708, hsa_circ_0032222, hsa_circ_0059662, and hsa_circ_0041685) and five downregulated (hsa_circ_0131433, hsa_circ_0123103, hsa_circ_0112036, hsa_circ_0113001, and hsa_circ_0050840) circRNAs (Figures 5A,B). The data showed that the levels of differentially quantified circRNAs by qRT-PCR were consistent with the results from microarray, supporting its reliability.

### Predicted circRNA-miRNA-mRNA Networks of Differentially Quantified circRNAs

As one of the mechanisms regulating gene expression, circRNA sponges miRNA and represses the effects of miRNA on mRNA. The miRNAs predicted to be sponged by 198 differentially quantified circRNAs formed an intricate network (Figure 5C). To explore the potential pathways involved with the differentially quantified circRNAs, verified differentially quantified circRNAs, including five upregulated (hsa_circ_0066867, hsa_circ_0093708, hsa_circ_0032222, hsa_circ_0059662, and hsa_circ_0041685) and five downregulated (hsa_circ_0131433, hsa_circ_0123103, hsa_circ_0112036, hsa_circ_0113001, and hsa_circ_0050840) circRNAs, were analyzed. Figure 5D showed the predicted network of circRNA–miRNA–mRNA, suggesting that verified differentially quantified circRNAs could regulate the expression of various target genes by sponging multiple miRNAs.

### GO and KEGG Pathway Analyses of Target Genes of Differentially Quantified circRNAs

**FIGURE 6:**
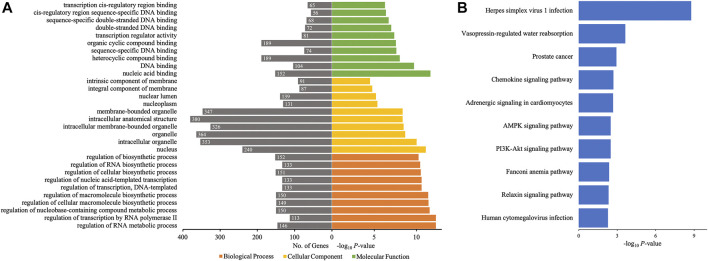
GO annotations and KEGG pathway analyses for target genes of the 10 differentially quantified circRNAs verified by qRT-PCR. **(A)** GO annotations. The GO analysis categorized the mRNAs into different groups under the theme of biological process, cellular component, and molecular function. The gene count for each GO category is listed on the left. **(B)** Top 10 KEGG pathways. The vertical axis indicates the pathway category, and the horizontal axis indicates the −log_10_ *p*-value of a pathway (Fisher’s exact test).

Then, GO and KEGG pathway analyses of target genes of 10 differentially quantified circRNAs verified by qRT-PCR were performed to study the potential roles of these circRNAs in AIS. As to the cellular component of GO annotation, the target genes were enriched in the nucleus (nucleus, nucleoplasm, and nuclear lumen), organelle (intracellular/intracellular membrane-bounded/membrane-bounded organelle), and membrane (intrinsic/integral component of membrane) (Figure 6A). The molecular functions were mainly related to DNA binding and *cis*-regulatory region, and the biological processes were associated with the regulation of macromolecules and nucleic acids (RNA and DNA). The KEGG analysis showed that metabolic (AMPK signaling pathway and PI3K-Akt signaling pathway), endocrine-related (vasopressin-regulated water reabsorption, adrenergic signaling in cardiomyocytes, and relaxin signaling pathway), and inflammatory (chemokine signaling pathway) pathways were enriched (Figure 6B).

According to circRNA–miRNA–mRNA networks, by GO and KEGG analyses, several differentially quantified circRNAs verified by qRT-PCR were involved in processes and pathways related to AIS. Hsa_circ_0112036 sponging miR-24-3p and regulating the levels of STRADB/BCL2L11/CDKN1B and hsa_circ_0066867 impacting miR-182-5p-FOXO3 were predicted in the AMPK and PI3K-Akt pathways. Hsa_circ_0093708, *via* miR-4269-CREB5 or miR-4533-AQP4, and hsa_circ_0041685, *via* miR-4267-DCTN2, played roles in the vasopressin-regulated water reabsorption. Moreover, hsa_circ_0066867 or hsa_circ_0041685 potentially regulated chemokine signaling through miR-6737-5p-CCL2 or miR-3192-5p-CXCL12, respectively.

### Coding Probability of Differentially Quantified circRNAs

**FIGURE 7:**
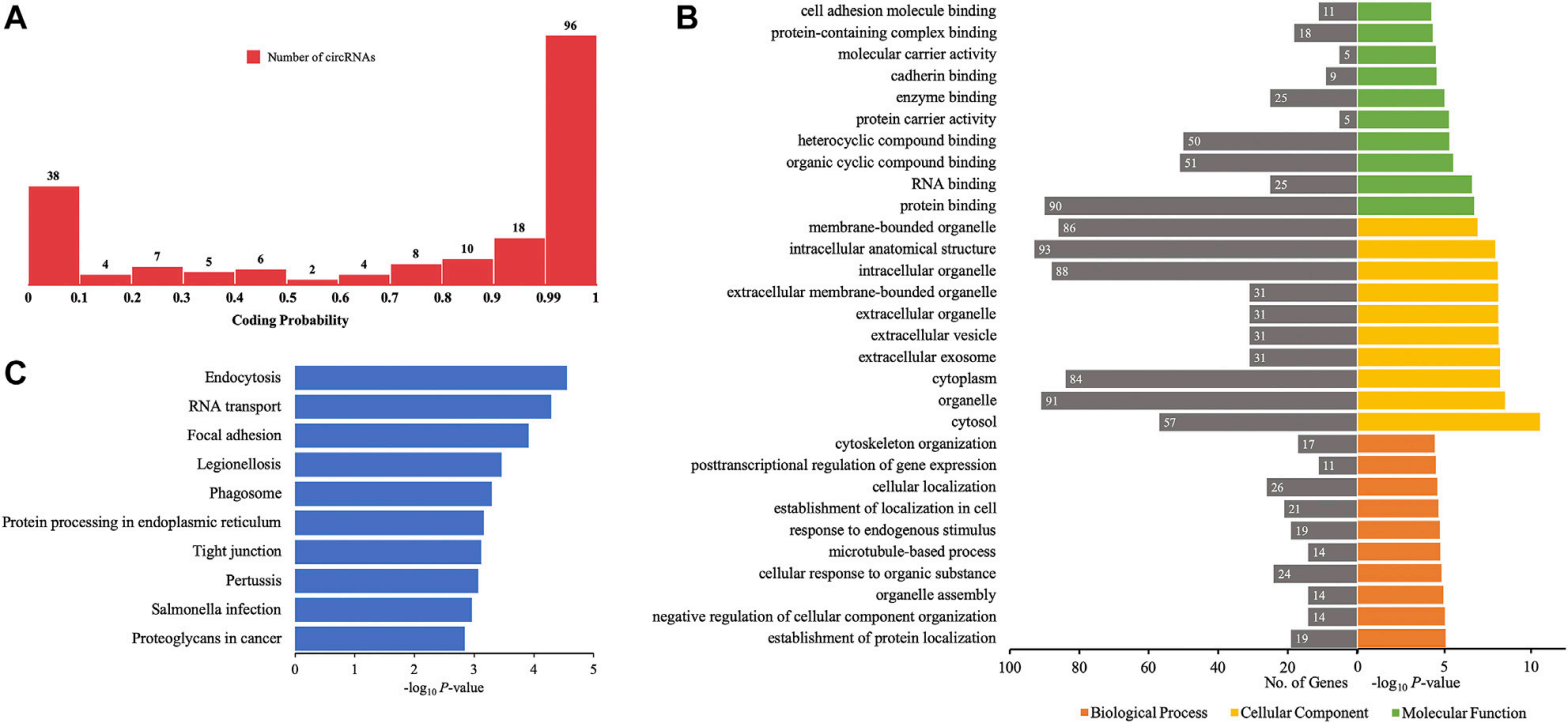
GO annotations and KEGG pathway analyses for proteins potentially coded by differentially quantified circRNAs. **(A)** Distribution of the coding probability of differentially quantified circRNAs. **(B)** GO annotations. The GO analysis categorized the genes into different groups under the theme of biological process, cellular component, and molecular function. The gene count for each GO category is listed on the left. **(C)** Top 10 KEGG pathways. The vertical axis indicates the pathway category, and the horizontal axis indicates the −log_10_ *p*-value of a pathway (Fisher’s exact test).

Certain circRNAs could be translated into proteins and participate in many processes of diseases. Thus, the coding probability of the differentially quantified circRNAs was analyzed. Of all 198 differentially quantified circRNAs, 96 circRNAs had strong coding potential (coding probability > 0.99), indicating that these molecules, through translation, could contribute to the process of AIS (Figure 7A).

To further investigate the role of proteins translated from differentially quantified circRNAs with strong coding potential in AIS, GO, and KEGG pathway analyses for these proteins were performed. The results of GO analysis showed that cellular components were mainly located in the intracellular (cytoplasm and organelle) and extracellular (vesicle and exosome) compartments. The molecular functions of these proteins were related to RNA and protein binding (enzyme/cadherin/cell adhesion molecule binding), and the biological processes were mainly related to localization (protein and cell) and cellular homeostasis (cellular component organization, organelle assembly, microtubule-based process, and cytoskeleton organization) (Figure 7B). The KEGG pathway analysis showed that intracellular activities (endocytosis, RNA transport, phagosome, and protein processing in endoplasmic reticulum) and interactions between cells (focal adhesion and tight junction) could be affected by proteins predicted to be translated from circRNAs (Figure 7C). Of the 10 verified differentially quantified circRNAs, hsa_circ_0041685 was predicted to be translated into RABEP1 and to impact the process of endocytosis.

### Prediction of RBPs Binding to Differentially Quantified circRNAs

**FIGURE 8:**
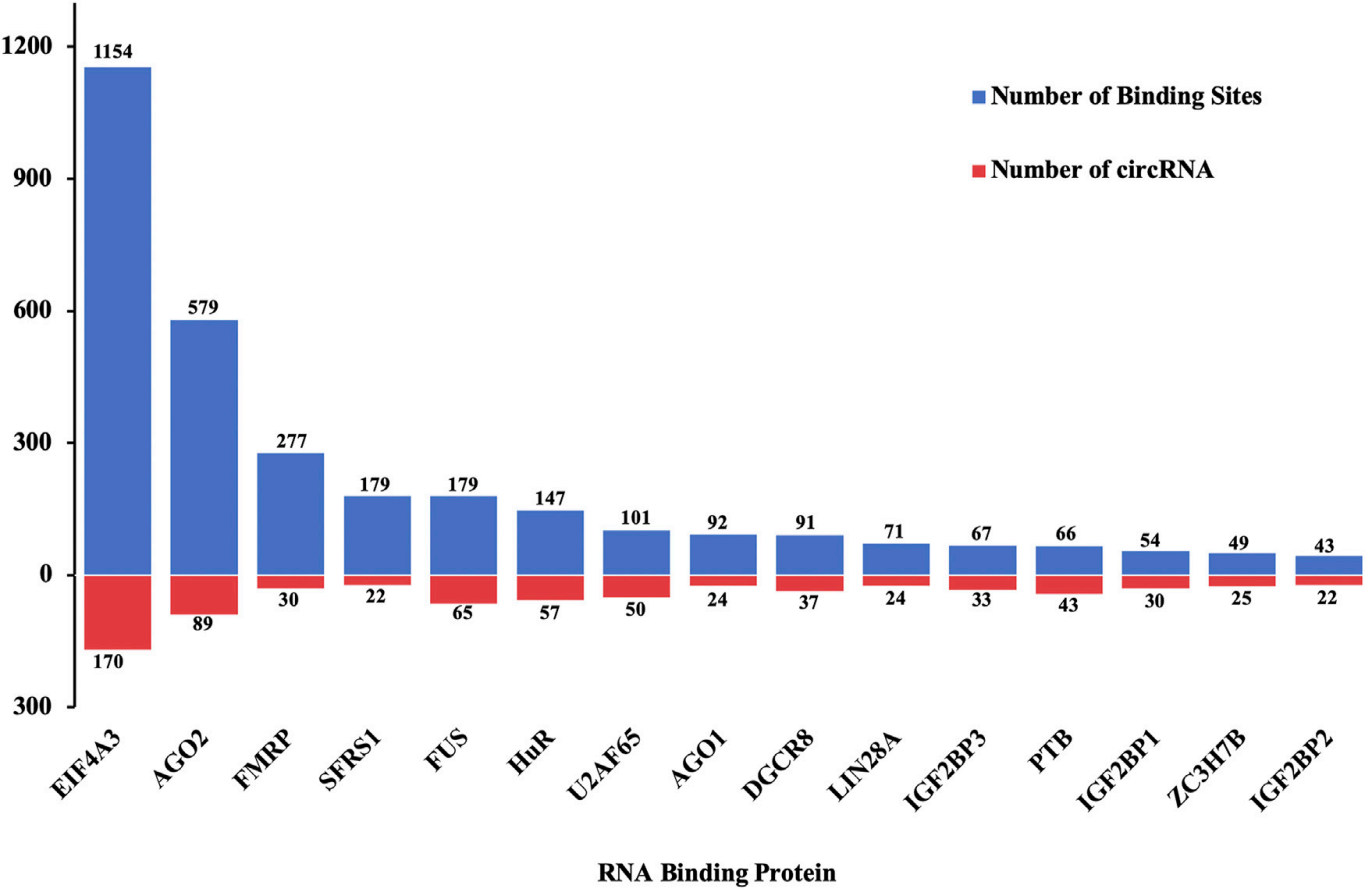
Distribution of top 15 RBPs predicted to bind to differentially quantified circRNAs.

Besides sponging miRNAs, circRNAs could bind to RBPs to regulate the expression of genes and processes of diseases. Among 38 RBPs, eukaryotic initiation factor 4A-3 (EIF4A3) had the highest count of binding sites for the differentially quantified circRNAs, while Argonaute 2 (AGO2) and fragile X mental retardation protein (FMRP) ranked second and third places, respectively. Moreover, of 198 differentially quantified circRNAs, 170 circRNAs were predicted to bind to EIF4A3, 89 circRNAs to bind to AGO2, and 65 circRNAs to bind to fused in sarcoma (FUS) (Figure 8).

### Evaluation of Diagnostic Value of circRNAs in AIS With ROC Curve Analysis

**FIGURE 9:**
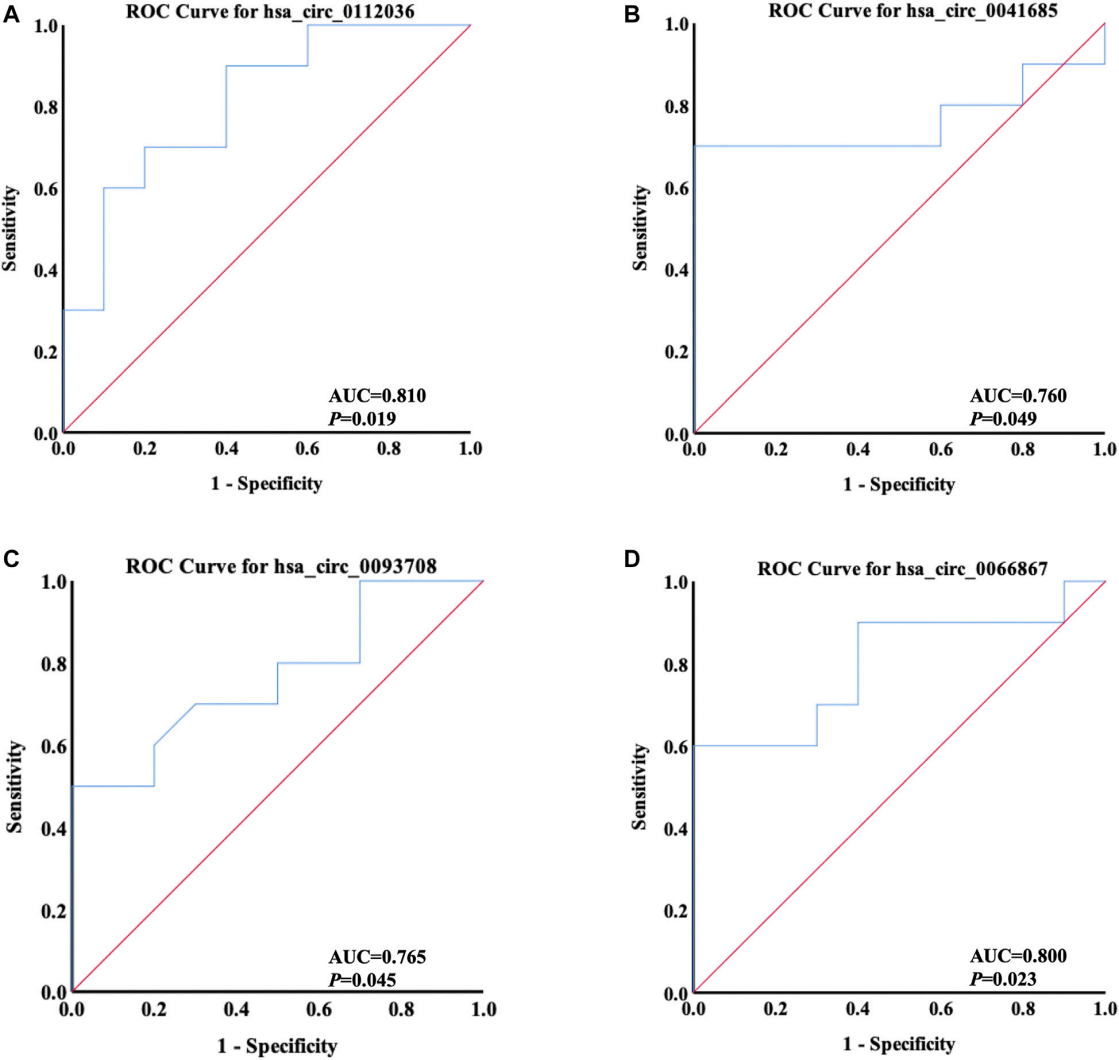
ROC curves of four differentially quantified circRNAs verified by qRT-PCR, including **(** **A** **)** hsa_circ_0112036, **(** **B** **)** hsa_circ_0041685, **(** **C** **)** hsa_circ_0093708, and **(** **D** **)** hsa_circ_0066867 (*n* = 10). The red diagonal lines in the graphs represent the diagnostic threshold value of circRNAs for AIS (AUC = 0.500).

ROC curve analysis was applied to evaluate the potential diagnostic value of the differentially quantified circRNAs derived from plasma exosomes. Based on the results of GO and KEGG analyses above, hsa_circ_0112036, hsa_circ_0066867, hsa_circ_0093708, and hsa_circ_0041685 were chosen for this analysis. The AUC of these four circRNAs ranged from 0.760 to 0.810 (*p* < 0.05), with the highest AUC found for hsa_circ_0112036 (AUC = 0.810) (Figure 9).

## Discussion

Stroke causes high mortality and disability rates globally (Hu et al., 2017). As the most common type of stroke, IS could be the result of various factors or diseases, including advanced age, smoking, hypertension, hyperlipidemia, and diabetes mellitus (Guzik and Bushnell, 2017). It has been reported that circRNA profiles of peripheral blood and peripheral blood mononuclear cells from patients with AIS were markedly different from those of controls (Li S. et al., 2020; Dong et al., 2020), indicating that circRNAs could be regulatory factors in AIS. However, it remains unknown whether circRNAs carried by exosomes mediate the signaling transduction and participate in the occurrence and progression of AIS. Hence, the present study aimed to elucidate the profile and potential function of circRNAs derived from plasma exosomes of patients with AIS.

By microarray, 198 differentially quantified circRNAs were found between patients with AIS and controls, evidencing that the profile of circRNAs derived from plasma exosomes was affected by AIS. CircRNAs are highly conserved and characterized by a stable structure and distinct tissue-specific expression (Memczak et al., 2013; Beermann et al., 2016). A tissue-specific circRNA database showed that the major origins of differentially quantified circRNAs with tissue specificity included the digestive system, the cardiovascular system, and lungs. The diseases contributing to AIS, like hypertension, atherosclerosis, and diabetes mellitus, cause marked impairment of the cardiovascular system, disrupting the expression of circRNAs (Wu et al., 2017; Zhao et al., 2020; Fu and Sun, 2021). The aberrant profile of circRNAs specific to the digestive system could be in part the result of the eating habits of patients with AIS. According to the latest literature, a high-fat diet could impair the colonic epithelium and alter the metabolic capacity of the microbiota, increasing the level of circulating trimethylamine N-oxide and the risk of atherosclerosis (Yoo and Zieba, 2021), which could partially explain the changes in the profile of circRNAs specific to the digestive system in AIS. Moreover, changes in the expression of lung-specific circRNAs could be contributing to the altered function of platelets in AIS. Platelet activation and platelet–leukocyte aggregation are independent determinants for AIS (Schmalbach et al., 2015). The lungs have been identified as a primary site of terminal platelet production and an organ with considerable hematopoietic potential (Lefrançais et al., 2017). This suggests that dysfunctional platelet from the lung could partially account for the association between aberrant expression of pulmonary circRNAs and AIS. These results indicate that, except for the well-known cardiovascular system, the digestive system and lungs could be non-negligible aspects in the pathogenesis of AIS.

Eukaryotic circRNAs are most commonly generated by back-splicing from pre-mRNAs of host genes—a process in which a downstream 5ʹ splice site joins with an upstream 3ʹ splice site—and regulate their expression *via* feedback (Memczak et al., 2013; Ashwal-Fluss et al., 2014; Lasda and Parker, 2014; Ragan et al., 2019). Thus, the profile of exosomal circRNAs could be an indicator of the expression profile of host genes. According to GO annotations of host genes, the ribosome and ribonucleoprotein could be impacted in AIS, disordering RNA translation/regulation and protein processing in the endoplasmic reticulum, which was highlighted by KEGG analysis. It has been reported that endoplasmic reticulum stress induced by abnormal protein processing takes place in atherosclerosis (Tabas, 2010; Huang et al., 2018) and hypertension (Naiel et al., 2019; Liu et al., 2020), for which the underlying mechanism could be the dysfunction of vascular endothelial cells by endoplasmic reticulum stress, like excessive apoptosis (Sun et al., 2015; Carlisle et al., 2016). Moreover, by KEGG pathway analysis, focal adhesion and leukocyte transendothelial migration were significantly enriched. Focal adhesions—contact points for the cell with the extracellular matrix—regulate communication of the cell with the surrounding extracellular environment and signaling of diverse cellular processes, including proliferation, migration, apoptosis, spreading, and differentiation (Carragher and Frame, 2004), also found in patients with large-artery atherosclerotic stroke by Xiao et al. (2021). There are a variety of molecules associated with focal adhesion, such as focal adhesion kinase (FAK), integrin, talin, and vinculin, and their activities are related to the development of hypertension (Sugimura et al., 2010; Sen et al., 2011; Jia et al., 2017), atherosclerosis (von Essen et al., 2016; Murphy et al., 2019), and thrombosis (Hitchcock et al., 2008), contributing to the progression of AIS. Moreover, leukocytes, especially monocytes, migrate from blood to the subendothelial space and form foam cells with lipoproteins, a key point to aggravate inflammation of the atherosclerotic plaque (Hansson and Libby, 2006; Gisterå and Hansson, 2017). Based on the data mentioned above, the diseases or factors causing AIS, including hypertension, atherosclerosis, and thrombosis, were significantly accompanied by altered profiles of host genes and circRNAs, demonstrating that circRNAs derived from plasma exosomes could be useful biomarkers to evaluate AIS or diseases/factors related to it. In addition, the data on GO and KEGG pathway analyses for host genes of differentially quantified circRNAs were different from those of previous research on blood of patients with AIS (Li S. et al., 2020), indicating that the profile of circRNAs from diverse origins was markedly distinct.

Though the function of most circRNAs remains unknown, a few circRNAs described in physiological and pathological processes generally act as miRNA/protein sponges or translate into proteins (Hansen et al., 2013; Zheng et al., 2016; Pamudurti et al., 2017). To evaluate the function of differentially quantified circRNAs found in the present study, by *in silico* analysis, the putative roles of exosome-derived circRNAs were predicted in AIS.

As miRNA sponges, circRNAs bind to miRNAs and consequently inhibit their function, which is a new mechanism in the regulation of miRNA activity and gene expression (Hansen et al., 2013; Memczak et al., 2013). In the present study, the interaction between differentially quantified circRNAs and miRNAs formed a complex network, suggesting that circRNAs could regulate various stages of AIS *via* sponging miRNAs. MiR-382-5p, containing binding sites for hsa_circ_0069594 and hsa_circ_0128924, is involved in the NFIA signaling regulating cholesterol homeostasis and inflammation (Hu et al., 2015). MiR-92a (potentially sponged by hsa_circ_0024722, hsa_circ_0059965, and hsa_circ_0062949) and miR-221/222 (potentially sponged by hsa_circ_0139214 and hsa_circ_0108959) participated in the course of atherosclerosis by targeting KLF2 mRNA, which modulates shear stress genes (Wu et al., 2011), and sustaining plaque stability (Bazan et al., 2015), respectively. Furthermore, miR-298 (potentially sponged by hsa_circ_0097102 and hsa_circ_0014293) exacerbated cerebral ischemia/reperfusion injury by targeting ACT1 mRNA (Sun et al., 2018), while miR-210 (potentially sponged by hsa_circ_0140265 and hsa_circ_0040760) targets CASP8AP2 mRNA and ISCU1/2 mRNA to suppress apoptosis and improve cerebral injury following AIS (Chan et al., 2009; Kim et al., 2009).

To explore the function of genes regulated by the circRNA–miRNA–mRNA interaction network, we performed GO and KEGG pathway analyses for the target genes of 10 differentially quantified circRNAs verified by qRT-PCR, including five upregulated (hsa_circ_0066867, hsa_circ_0093708, hsa_circ_0032222, hsa_circ_0059662, and hsa_circ_0041685) and five downregulated (hsa_circ_0131433, hsa_circ_0123103, hsa_circ_0112036, hsa_circ_0113001, and hsa_circ_0050840) circRNAs. The results showed that, as competing endogenous RNAs, differentially quantified circRNAs, through misregulating RNA and DNA, would impact cellular components, including the nucleus, organelle, and membrane, and modify the function and fate of cells/tissues. AIS induces neuron apoptosis (Jiang et al., 2018), while autophagy could rescue neurons from apoptosis and confer neuroprotection in AIS (Wang et al., 2012; Wang et al., 2014). These processes involved organelles, like the mitochondria and endoplasmic reticulum (Chen et al., 2002; Yorimitsu et al., 2006; Heath-Engel et al., 2008; Guan et al., 2018), and signaling pathways enriched by KEGG analysis, including PI3K-Akt and AMPK pathways (Sheng et al., 2010; Jiang et al., 2015; Hou et al., 2018; Wang et al., 2020). Similarly, Li et al. 2020b also predicted that circRNAs from the blood could regulate apoptosis in AIS. Based on the circRNA–miRNA–mRNA network, hsa_circ_0112036 and hsa_circ_0066867 have been predicted to regulate mRNAs related to AMPK and PI3K-Akt signaling, like STRADB, BCL2L11, CDKN1B, and FOXO3, and be involved with the process of AIS. Moreover, KEGG analysis highlighted pathways related to vasopressin-regulated water reabsorption and chemokine signaling. It has been reported that vasopressin is associated with stroke-related edema (Ameli et al., 2014) and that aquaporin 4 (AQP4), the most ubiquitous water channel in the central nervous system and abundantly expressed in astrocytes, participates in the vasopressin-regulated water reabsorption. Zheng et al. 2017 reported that astrocyte cell injury would be ameliorated by downregulation of AQP4 expression in cerebral IS. In the present study, the hsa_circ_0093708-miR-4533-AQP4 axis was predicted, and the increased level of hsa_circ_0093708 in plasma exosomes of AIS patients suggested that AIS patients may have upregulated the expression of AQP4 and, therefore, be prone to brain injury. Chemokine signaling was indispensable for leukocyte migration, which is a crucial event in the development of inflammation and atherosclerosis (Ruytinx et al., 2018; Yan et al., 2021). In this context, we found that hsa_circ_0066867 and hsa_circ_0041685, significantly increased in AIS patients, potentially regulate chemokine signaling through miR-6737-5p-CCL2 and miR-3192-5p-CXCL12, respectively. Previous studies showed that CCL2 promoted IS *via* chemokine signaling (Li L. et al., 2020) and that the level of serum CXCL12 was positively correlated with stroke severity (Liu et al., 2015). Therefore, circRNAs from plasma exosomes could potentially prevent and/or assist in the treatment of AIS. Besides, the results on target genes of differentially quantified circRNAs were different from those of Dong et al. (2020), in which circRNAs of peripheral blood mononuclear cells from patients with AIS were involved in inflammation and immunity. We speculated that, compared with circRNAs from a single source, circRNAs from plasma exosomes originating from various cells/tissues could provide more comprehensive information as to AIS.

Since researchers found that certain proteins translated by circRNAs participate in the processes of human diseases (Yang et al., 2018; Zhang et al., 2018), a variety of circRNAs have been described as coding RNAs, and the resulting proteins may play biological roles in the emergence and progression of human diseases. The translational potential of differentially quantified circRNAs were assessed, and the functions of circRNA-translated proteins were also analyzed by GO and KEGG. We found that 96 of 198 differentially quantified circRNAs had strong translational potential. On one hand, the proteins that might be translated from differentially quantified circRNAs with translational potential may contribute to intracellular metabolic processes and functions of cells, including RNA binding, organelle assembly, microtubule-based process, and protein processing in the endoplasmic reticulum. On the other hand, these proteins were involved in the communication between cells *via* several ways, like endocytosis, phagosome, focal adhesion, and tight junction. Endocytosis—a ubiquitous physiological process mediating nutrient uptake, receptor internalization, and signaling, essential events for cell growth and survival—has been reported to cause neuronal death and exacerbate brain damage during AIS (Troulinaki and Tavernarakis, 2012; Tejeda et al., 2019; Diaz-Guerra, 2021). RABEP1 is an essential and rate-limiting component of the endosome fusion regulating early endosomal transport in endocytosis. Upregulated hsa_circ_0041685 might result in increased level of RABEP1 and accelerate endocytosis, playing a role in the development of AIS. Focal adhesion, predicted by GO and KEGG analyses of host genes as well, played an important role in various inducers of AIS, including hypertension (Jia et al., 2017), atherosclerosis (von Essen et al., 2016), and thrombosis (Hitchcock et al., 2008). Chen et al. reported that, in a mouse model, targeting of FAK could be a potential treatment for early IS (Chen et al., 2018). Tight junction is a selectively permeable barrier that generally represents the rate-limiting step of paracellular transport, and, as one of the pathophysiological features of AIS, loss of blood–brain barrier tight junction integrity results in vasogenic edema, hemorrhagic transformation, and increased mortality (Abdullahi et al., 2018; Yang et al., 2019). Thus, we suspected that exosome-derived circRNAs could be a new treatment approach to AIS. Furthermore, there were several events highlighted by GO and KEGG analyses of both host genes and proteins encoded by circRNAs, suggesting that disordered expression of host genes could affect the profile of circRNAs in AIS, and vice versa.

RBPs assemble the ribonucleoprotein complexes to bind RNA sequences by interacting with specific *cis*-regulatory elements and affect the expression and function of their target RNAs (Janga and Mittal, 2011). Increased evidence indicates that many circRNAs interact with RBPs (Hansen et al., 2013; Ashwal-Fluss et al., 2014; Abdelmohsen et al., 2017) and that the interactions between circRNAs and RBPs are also deemed to be an essential element involved in gene transcriptional regulation (Hansen et al., 2013; Memczak et al., 2013), circRNA translation (Yang et al., 2017), and RBP sponging (Du et al., 2017b). Therefore, the distribution of putative RBPs interacting with differentially quantified circRNAs was investigated in the present study. The data showed that the main RBPs binding to differentially quantified circRNAs were EIF4A3 and AGO2. EIF4A3, a core component of the exon junction complex, regulated neuronal cell injury by targeting cGLIS3 in AIS (Jiang et al., 2021). AGO2 is a critical component of the RNA-induced silencing complex and, as a consequence, a master regulator of miRNA-dependent gene silencing. AGO2-associated miRNA profiles were modified in the brain of a rat stroke model (Liu X. S. et al., 2017). Besides, RBPs can be critical factors for the promotion of circRNA transmission from cells (Janas et al., 2015) and can serve as intracellular inducers of circRNA loading in exosomes (O'Leary et al., 2017), which could assist circRNAs in mediating communication between cells *via* exosomes.

CircRNAs are stable and abundant and function as effective diagnostic biomarkers of stroke, like hsa_circ_0001599 (Li et al., 2021) and hsa_circ_0141720 (Chen Y. et al., 2020). In order to explore new circRNAs as potential biomarkers of AIS, ROC curves of four circRNAs (hsa_circ_0112036, hsa_circ_0066867, hsa_circ_0093708, and hsa_circ_0041685) predicted to play a role in the pathogenesis of AIS were analyzed. The data showed that the levels of the four circRNAs could significantly differentiate AIS patients from controls, with hsa_circ_0112036 possessing the highest AUC. These results indicated that the four circRNAs could be further explored as promising biomarkers for AIS diagnosis.

Taken together, the data of this study demonstrate that circRNAs derived from plasma exosomes were differentially quantified between AIS patients and controls. Importantly, these results revealed that exosomal circRNAs from plasma, especially hsa_circ_0112036, hsa_circ_0066867, hsa_circ_0093708, and hsa_circ_0041685, potentially participate in the progression of AIS *via* sponging miRNA/RBPs or encoding proteins, may be explored as biomarkers for the diagnosis of AIS, and may also be potential targets for therapeutic interventions. However, these findings are based on only 10 AIS patients and 10 controls, and more samples are necessary to assess and validate the data in the future.

## Data Availability

The microarray datasets generated during the current study have been deposited in the NCBI Gene Expression Omnibus (GEO) repository and are accessible via GEO series accession number GSE195442 (https://www.ncbi.nlm.nih.gov/geo/query/acc.cgi?acc=GSE195442).
